# Supplementary material for: Mammographic Density and Screening Sensitivity, Breast Cancer Incidence and Associated Risk Factors in Danish Breast Cancer Screening
Source: J Clin Med. 2019 Nov 19;8(11):2021. doi: 10.3390/jcm8112021 (PMC6912479; doi:10.3390/jcm8112021)
Supplement: Supplementary file 1 [file jcm-08-02021-s001.pdf]

Supplementary Table 1. Data sources used in Danish studies on mammographic density.

Reference	Year and location	Type of density data	Study design	Risk factor studied	Risk factor data	Follow-up data	Screening outcome	Interval cancers	Outcome measure
Studies on sensitivity/specificity by density									
Olsen et al, 2009 (4)	1991-2001 Copenhagen	Fatty and Mixed/dense	Cohort to 2001	NA	NA	Population register	Screening program database	Danish Cancer Register	Sensitivity
Von Euler-Chelpin et al, 2018 (7)	2012-2013 Capital Region	BI-RADS, version 4	Cross sectional	NA	NA	NA	Screening program database	Danish Cancer Register	Sensitivity, specificity
Study on prevalence of fatty and of mixed/dense breast by birth cohort									
Hellmann et al, 2013 (5)	1991-2001 Copenhagen and Funen county	Fatty and Mixed/dense	Time trend	Hormone therapy	Drug Prescription database	NA	NA	NA	Prevalence of fatty and mixed/dense
Studies on risk factors for mixed/dense breasts									
Azam et al, 2018 (15)	1993-1997 Copenhagen	Fatty and Mixed/dense	Cross sectional	Physical activity	Diet, Cancer, Health cohort	NA	NA	NA	Odds ratio for mixed/dense exposed vs non-exposed
Azam et al, 2018 (12)	1993-1997 Copenhagen	Fatty and Mixed/dense	1)Cross sectional for density 2)Cohort for breast cancer to 2010	Hormone therapy	Diet, Cancer, Health cohort	Population register	Breast cancer incidence data: Danish Cancer Register		1)Odds ratio for mixed/ dense 2)Hazard ratio for incident breast cancer
Roswall et al, 2018 (16)	1993-1997 Copenhagen	Fatty and Mixed/dense	Cross sectional	Traffic noise	Modelled traffic noise 5 years before mammogram	NA	NA	NA	Odds ratio for mixed/dense per quartile of exposure
Jacobsen et al, 2017 (17)	1993-1997 Copenhagen	Fatty and Mixed/dense	Cross sectional	Alcohol consumption	Diet, Cancer, Health	NA	NA	NA	Odds ratio for mixed/dense exposed vs non-exposed

Buschard et al, 2017 (13)	1993-1997 Copenhagen	Fatty and Mixed/dense	Cross sectional	Diabetes	Diet, Cancer, Health	NA	NA	NA	Odds ratio for mixed/dense exposed vs non-exposed
Jacobsen et al, 2016 (14)	1993-1997 Copenhagen	Fatty and Mixed/dense	Cross sectional	Cigarette smoking	Diet, Cancer, Health	NA	NA	NA	Odds ratio for mixed/dense exposed vs non-exposed
Huynh et al, 2015 (18)	1993-1997 Copenhagen	Fatty and Mixed/dense	Cross sectional	Air pollution	Modelled nitrogen oxide 1971-1993/7	NA	NA	NA	Odds ratio for mixed/dense per 20 μg/m^3 of exposure
Studies on breast cancer incidence by density									
Olsen et al, 2009 (4)	1991-2001 Copenhagen	Fatty and Mixed/dense	Cohort to 2001	NA	NA	Population register	Breast cancer incidence Danish Cancer Register		Rate ratio for breast cancer, mixed/dense vs fatty
Von Euler-Chelpin et al, 2018 (7)	2012-2013 Capital Region	BI-RADS version 4	Cross sectional	NA	NA	NA	Screening program database	Danish Cancer Register	Relative risk for breast cancer, baseline BI-RADS 1
Andersen et al, 2014 (10)	1991- Copenhagen	Fatty and Mixed/dense	Cohort to 2009	Childhood body constitution	School Health register	Population register	Breast cancer incidence Danish Cancer register		Rate ratio for breast cancer by body constitution controlled for density
